# Drought severity determines the effectiveness of straw mulching in alleviating water stress during early maize growth

**DOI:** 10.3389/fpls.2026.1983668

**Published:** 2026-10-22

**Authors:** Renjie Zhao, Zexin Qi, Lihua Zhang, Fei Li, Ning Sun, Weiping Yan, Mo Zhu, Qian Li, Hongxiang Zhao, Chen Xu, Zhian Zhang

**Affiliations:** 1 Jilin Academy of Agricultural Sciences (Northeast Agricultural Research Center of China)/Key Laboratory of Water-Saving Agriculture in Northeast China (Jilin), Ministry of Agriculture and Rural Affairs, Changchun, China; 2 Baicheng, Multidisciplinary, Observation and Research Station, Ministry of Agriculture and Rural Affairs, Taonan, China; 3 Key Laboratory of Molecular Epigenetics of Ministry of Education, Northeast Normal University, Changchun, China; 4 Agronomy College, Jilin Agricultural University, Changchun, China

**Keywords:** carbon assimilation, drought severity, maize, oxidative stress, photosynthesis, straw mulching

## Abstract

**Introduction:**

Drought is a major constraint on the early growth and photosynthetic productivity of maize (Zea mays L.), yet whether the physiological benefits of straw mulching depend on drought severity remains unclear.

**Methods:**

A two-year experiment was conducted in 2025 and 2026 using straw removal and straw mulching under three irrigation levels (135, 105, and 75 mm), representing well-watered, moderate-drought, and severe-drought conditions, respectively. Plant growth, photosynthetic performance, carbon-assimilation enzyme activities, osmotic adjustment, and antioxidant responses were evaluated.

**Results:**

Increasing drought severity generally reduced leaf area index (LAI), plant height (PH), dry matter accumulation (DW), photosynthetic performance, and carbon-assimilation capacity, while increasing the accumulation of proline, soluble sugars, betaine, malondialdehyde (MDA), and hydrogen peroxide (H_2_O_2_), together with antioxidant responses. The physiological effects of straw mulching varied markedly with drought severity. Under moderate drought, mulching more consistently maintained plant growth and photosynthetic performance, preserved carbon-assimilation enzyme activities, and reduced MDA and H₂O₂ accumulation. In contrast, these beneficial responses were substantially weaker and less consistent under severe drought. Across physiological traits, better growth and carbon-assimilation performance were generally associated with lower osmotic and oxidative stress. Multivariate patterns further showed that mulched plants under moderate drought exhibited a physiological profile closer to that under well-watered conditions, whereas plants under severe drought remained distinctly separated despite straw mulching.

**Discussion:**

Overall, straw mulching provided more consistent physiological protection against moderate drought than severe drought during early maize growth, indicating that drought severity strongly constrains the effectiveness of straw mulching.

## 1 Introduction

Maize (*Zea mays* L.) is one of the most important cereal, feed, and industrial crops worldwide, and stable maize production is essential for food security. However, uneven precipitation distribution and increasingly frequent seasonal drought have made water deficit a major environmental constraint on maize growth and productivity (Singh et al., 2023). The effects of drought on crops depend strongly on the developmental stage, duration, and severity of stress, and different levels of water deficit can induce markedly different growth and physiological responses (Farooq et al., 2009). During vegetative growth, water limitation inhibits cell division and expansion in maize, thereby restricting leaf expansion, plant height, and dry matter accumulation, and these inhibitory effects become progressively stronger with increasing drought severity (Avramova et al., 2015). Because early vegetative growth is a critical period for rapid canopy establishment and the development of photosynthetic capacity, alleviating water stress during this stage is important for maintaining subsequent crop growth and assimilate production.

Photosynthesis is among the physiological processes most sensitive to water deficit. During the early stages of drought, stomatal closure reduces transpirational water loss but simultaneously restricts CO_2_ diffusion into the leaf, resulting in decreases in stomatal conductance and net photosynthetic rate. As water deficit becomes more severe, mesophyll conductance, photochemical processes, and carbon metabolism can also be inhibited, shifting photosynthetic limitation from predominantly stomatal control toward a combination of stomatal and non-stomatal limitations (Chaves et al., 2009). Although maize is a typical C4 crop with an efficient CO_2_-concentrating mechanism, its photosynthetic apparatus remains sensitive to water deficit, and severe drought can impair carbon-assimilation enzyme activity and photosynthetic metabolism (Ghannoum, 2009). Drought also disturbs the balance between the production and scavenging of reactive oxygen species (ROS), resulting in the excessive accumulation of superoxide radicals and H_2_O_2_ and subsequently inducing membrane lipid peroxidation and cellular damage (Mittler, 2002; Asada, 2006). Plants counteract these effects by accumulating compatible osmolytes, including proline, soluble sugars, and betaine, and by activating antioxidant enzymes such as superoxide dismutase, peroxidase, and catalase (Farooq et al., 2009; Avramova et al., 2015). Thus, plant growth, photosynthetic carbon assimilation, osmotic adjustment, and antioxidant defense constitute an interconnected physiological network that determines maize adaptation to drought stress.

Straw mulching is an important surface-management practice in conservation agriculture, with one of its major functions being the regulation of the soil hydrothermal environment. Early studies demonstrated that maintaining crop residues on the soil surface can reduce soil evaporation, although maize responses to mulching depend substantially on irrigation regime, soil type, and environmental conditions (Tolk et al., 1999). In dryland cropping systems in China, straw mulching has been shown to reduce non-productive soil evaporation, increase soil water storage, and improve water use efficiency (Liu et al., 2010; Li et al., 2013). Long-term field experiments further demonstrated that appropriate straw mulching can improve soil water storage, water use efficiency, and spring maize productivity, although these effects vary with mulch amount and application pattern (Cai et al., 2015; Lin et al., 2016). In addition to conserving soil water, straw mulching can modify the soil–plant microclimate by reducing soil evaporation and buffering fluctuations in soil temperature and moisture, thereby creating a more favorable root-zone environment for plant water uptake and physiological activity. These microclimatic improvements can help sustain leaf expansion and photosynthetic performance and ultimately promote aboveground biomass accumulation in maize under water-limited conditions (Zhang et al., 2019). Nevertheless, the effects of straw mulching on maize are not universally positive across environments. In the Mollisol region of Northeast China, straw mulching increased soil moisture under relatively adequate water supply but reduced early-season soil temperature, which could suppress maize growth, water use efficiency, and grain yield (Lu et al., 2015). A subsequent meta-analysis showed that the effects of straw mulching on maize yield and water use efficiency are strongly regulated by precipitation, evaporation, temperature, soil texture, and straw application rate, with greater benefits generally occurring under water-limited environments (Qin et al., 2021). More recently, a multi-year experiment in the semi-arid region of Northeast China showed that the positive effect of straw mulching on maize grain yield was greater in years with lower rainfall and diminished as precipitation increased (Nie et al., 2026). Collectively, these findings demonstrate that the effectiveness of straw mulching is strongly dependent on environmental conditions, particularly water availability, rainfall distribution, and the timing of mulch application relative to the occurrence of drought stress. Previous studies have shown that the benefits of mulching may be reduced when rainfall patterns are highly variable or when the timing of mulch application does not coincide with critical periods of crop water demand, indicating that its effectiveness is closely linked to both stress intensity and stress timing (Singh et al., 2023). These observations suggest that the drought-mitigating capacity of straw mulching may operate within a defined range of environmental conditions rather than providing uniform protection across all levels of water deficit. However, compared with the extensive information available on soil water status, water use efficiency, and final maize yield, considerably less is known about whether straw mulching can maintain photosynthetic carbon assimilation, osmotic homeostasis, and antioxidant capacity during early maize growth as drought severity increases, or whether these physiological benefits diminish once water deficit becomes severe.

Collectively, these findings demonstrate that the effectiveness of straw mulching is strongly influenced by environmental conditions, particularly water availability, rainfall distribution, and the timing of mulch application relative to drought occurrence. Previous studies have largely focused on changes in soil water status, water-use efficiency, and final crop productivity under straw mulching, whereas comparatively less attention has been paid to how the physiological effectiveness of mulching changes as drought severity increases. In particular, it remains unclear whether straw mulching can consistently maintain photosynthetic carbon assimilation, osmotic homeostasis, and antioxidant capacity across different levels of water deficit, or whether these physiological benefits diminish once drought becomes severe. Clarifying this drought-severity-dependent physiological response is essential for understanding the conditions under which straw mulching can effectively mitigate water stress during early maize growth.

Accordingly, the present study focused on the early growth stage of maize and compared straw removal and straw mulching under well-watered, moderate-drought, and severe-drought conditions. We systematically evaluated plant growth, leaf photosynthetic performance, carbon-assimilation enzyme activities, osmotic adjustment and membrane lipid peroxidation, and antioxidant defense, and further integrated these traits using multivariate analysis. We hypothesized that the physiological benefits of straw mulching would be more consistently expressed under moderate drought than under severe drought. This study therefore aimed to determine how drought severity influences the physiological effectiveness of straw mulching during early maize growth and to identify the physiological responses associated with its drought-mitigating effects.

## 2 Experimental design

### 2.1 Experimental site

The experiment was conducted at the Crop Water Use Efficiency Experimental Station of the Jilin Academy of Agricultural Sciences in Gongzhuling, Jilin Province, China (124°48′25″ E, 43°30′58″ N). The region has a northern temperate continental monsoon climate, with a frost-free period of approximately 144 d. During the 2025 maize growing season (May–October), the mean daily air temperature was 21.56 °C. Before the experiment, the 0–40 cm soil layer contained 22.40 g kg^-1^ organic matter, 1.54 g kg^-1^ total N, 42.10 mg kg^-1^ available P, and 120.30 mg kg^-1^ available K, with a pH of 6.53. The soil was classified as black soil.

### 2.2 Experimental design

The maize cultivar Fumin 985, which is widely grown in central and western Jilin Province, was used in this study. The cultivar has a growth duration of approximately 128 d. Field experiments were conducted for two consecutive growing seasons in 2025 and 2026, and maize was sown on April 28 in both years. A fully openable movable rainout shelter was installed at the experimental site. During rainfall events, the shelter could be completely closed using motors installed on both sides to exclude natural precipitation and ensure precise control of the imposed water treatments.

The experiment consisted of two factors: straw management and drought severity. Straw management included straw removal and straw mulching. The straw used for mulching was maize residue from the preceding crop. After maize harvest in autumn, the straw was chopped into pieces ≤10 cm in length and uniformly applied to the soil surface at a rate of 4,000 kg ha^-1^ (0.4 kg m^-^²) to achieve complete surface coverage. Before maize sowing in the following spring, the straw was repositioned to both sides of the planting rows to facilitate field operations while maintaining surface mulch. The mulching treatment was initiated after harvest in autumn 2024 and renewed after harvest in autumn 2025 following the same procedure. The irrigation treatments were established with reference to a previously developed whole-season irrigation regime for maize in the same region, in which 500, 350, and 200 mm represented normal irrigation, moderate-deficit irrigation, and severe-deficit irrigation, respectively (Qi et al., 2025). Because the present study focused on the early vegetative growth period rather than the entire maize growing season, the irrigation amounts were adjusted according to the water requirement during this stage. Accordingly, three irrigation levels were established: 135 mm (well-watered), 105 mm (moderate drought), and 75 mm (severe drought), with 135 mm corresponding approximately to the water requirement of maize during early vegetative growth under local climatic conditions. Six treatments were therefore established: CK1, straw removal with 135 mm irrigation; CK2, straw mulching with 135 mm irrigation; T3, straw removal with 105 mm irrigation; T4, straw mulching with 105 mm irrigation; T1, straw removal with 75 mm irrigation; and T2, straw mulching with 75 mm irrigation (**Table 1**).

**Table 1:** Experimental treatments.

Treatment	Straw management	Irrigation amount (mm)
T1	Straw removal	75
T2	Straw mulching	75
T3	Straw removal	105
T4	Straw mulching	105
CK1	Straw removal	135
CK2	Straw mulching	135

Irrigation was applied using a drip irrigation system. Drip lines were placed on the ridge approximately 5 cm from the maize planting row. The emitter spacing was 20 cm, the emitter discharge rate was 2.3 L h^-1^, and the drip line thickness was 0.25 mm. Irrigation was applied eight times during the treatment period, and the irrigation interval and amount per irrigation event were adjusted according to the designated irrigation level (**Table 2**).

**Table 2:** Drip irrigation schedule under different irrigation treatments.

Irrigation level (mm)	Irrigation events	Interval (d)	Irrigation amount per event (mm)
75	1st–2nd	15	15
	3rd–5th	10	10
	6th–8th	10	15
105	1st–2nd	15	20
	3rd–5th	10	15
	6th–8th	10	20
135	1st–2nd	15	25
	3rd–5th	10	20
	6th–8th	10	25

Maize was grown using a conventional ridge-cropping system. Each plot had an area of 24 m², with a plot length of 5 m and a row spacing of 0.60 m. The planting density was 60,000 plants ha^-1^. Treatments were arranged in a randomized complete block design with three replicates, resulting in a total of 18 plots. Each plot was separated from adjacent plots by cement walls along both the sides and bottom to minimize lateral and vertical water exchange and prevent interference from external soil water.

Before sowing, a compound fertilizer (N–P_2_O_5_–K_2_O, 24–14–16; Gongzhuling Difu Fertilizer Co., Ltd., Gongzhuling, China) was applied as a basal fertilizer at 750 kg ha^-1^. Other field management practices were consistent with local maize production practices throughout the growing season.

### 2.3 Measurements and methods

#### 2.3.1 Plant agronomic traits

At the sixth-leaf stage (V6), eighth-leaf stage (V8), and twelfth-leaf stage (V12), three maize plants with uniform growth were selected from each plot. The leaf area of each fully expanded leaf was determined using a ruler. After measurement, the sampled plants were separated into individual organs and placed in paper bags. The samples were heated at 105 °C for 30 min to terminate physiological activity and subsequently oven-dried at 80 °C to a constant weight. The dry matter accumulation per plant was then determined using an electronic balance.

#### 2.3.2 Leaf photosynthetic characteristics

At V6, V8, and V12, measurements were conducted on clear, cloudless mornings. Three maize plants with uniform growth were selected from each plot. Leaf gas-exchange parameters were measured using a LI-6400XT portable photosynthesis system (LI-COR Inc., Lincoln, NE, USA), including net photosynthetic rate (Pn), stomatal conductance (Gs), intercellular CO_2_ concentration (Ci), and transpiration rate (Tr). The stomatal limitation value (Ls) was calculated as Ls = (1 − Ci/Ca) × 100%, where Ca represents the CO_2_ concentration of the incoming air and was maintained at 400 μmol mol^−1^ during measurement. Apparent mesophyll conductance (AMC) was calculated as AMC = Pn/Ci. For all measurements, the second fully expanded leaf from the top of each selected maize plant was used. Water use efficiency (WUE) was calculated as the ratio of net photosynthetic rate to transpiration rate: WUE = Pn/Tr, where Pn is the net photosynthetic rate and Tr is the transpiration rate.

### 2.4 Activities of carbon metabolism-related enzymes in leaves

At V6, V8, and V12, three maize plants with uniform growth were selected from each plot, and the second fully expanded leaf from the top of each plant was sampled. Biochemical traits were determined using commercial enzyme-linked immunosorbent assay (ELISA) kits (Meimian Biotechnology Co., Ltd., Jinan, China) according to the manufacturer’s instructions. Briefly, leaf extracts were diluted with the supplied sample diluent, and 10 μL of sample was added to each well together with 40 μL of sample diluent. After incubation at 37 °C for 30 min, the plates were washed five times, followed by the addition of 50 μL enzyme-conjugate reagent and a second incubation at 37 °C for 30 min. After another washing step, 50 μL each of chromogenic solutions A and B were added, and the plates were incubated at 37 °C in the dark. The reaction was terminated with 50 μL stop solution, and absorbance was measured at 450 nm within 15 min using a microplate reader. Concentrations or activities were calculated from the corresponding standard curves and corrected for the sample dilution factor. The activities of ribulose-1,5-bisphosphate carboxylase (RuBPCase) and pyruvate carboxylase (PC) were determined using the corresponding ELISA kits.

### 2.5 Activities of carbon metabolism-related enzymes in leaves

At V6, V8, and V12, three maize plants with uniform growth were selected from each plot, and the second fully expanded leaf from the top of each plant was sampled. The activities of ribulose-1,5-bisphosphate carboxylase (RuBPCase) and pyruvate carboxylase (PC) were determined using commercial ELISA kits (Meimian Biotechnology Co., Ltd., Jinan, China) according to the procedure described above.

### 2.6 Contents of osmotic adjustment substances in leaves

At V6, V8, and V12, three maize plants with uniform growth were selected from each plot, and the second fully expanded leaf from the top of each plant was sampled. The contents of proline (Pro), soluble sugars (SS), betaine (BE), and malondialdehyde (MDA) were determined using commercial ELISA kits (Meimian Biotechnology Co., Ltd., Jinan, China) according to the procedure described above.

### 2.7 Leaf antioxidant characteristics

At V6, V8, and V12, three maize plants with uniform growth were selected from each plot, and the second fully expanded leaf from the top of each plant was sampled. The superoxide anion (O_2_^-^) scavenging rate, hydrogen peroxide (H_2_O_2_) content, and the activities of peroxidase (POD), catalase (CAT), and superoxide dismutase (SOD) were determined using commercial ELISA kits (Meimian Biotechnology Co., Ltd., Jinan, China) according to the procedure described above.

### 2.8 Data analysis

Experimental data were organized using Microsoft Excel, and statistical analyses were performed using SPSS 26.0. Statistical analyses were conducted separately for each experimental year (2025 and 2026) and each developmental stage (V6, V8, and V12). For each year and developmental stage, a two-way analysis of variance (ANOVA) was performed to evaluate the main effects of irrigation level (I), straw management (S), and their interaction (I × S). The individual plot was considered the experimental unit, with three replicate plots for each treatment. Three plants sampled within each plot were treated as subsamples, and their measurements were averaged to obtain a single plot-level value for statistical analysis. When significant treatment effects were detected, mean comparisons were performed using the least significant difference (LSD) test at P < 0.05. Data are presented as mean ± standard deviation (SD) of three replicate plots. Figures for growth, photosynthetic, physiological, and antioxidant traits were generated in R using the ggplot2 package (Wickham, 2016) and assembled with patchwork (Pedersen, 2024). Pearson correlation analysis and visualization were performed using the corrplot package (Wei and Simko, 2024). Principal component analysis (PCA) was performed using the prcomp function in R. For PCA, data from 2025 and 2026 were pooled. For each independent plot replicate, measurements obtained at V6, V8, and V12 were averaged for each variable before analysis. All variables were standardized to zero mean and unit variance (Z-score standardization) to eliminate differences in measurement scale. Each independent plot replicate for a given treatment and year constituted one observation in the PCA. PCA biplots were generated using ggplot2 (Wickham, 2016) and ggrepel (Slowikowski, 2024). Data are presented as mean ± standard deviation (SD). The main effects of irrigation level (I) and straw management (S), together with their interaction (I × S), were evaluated separately for each developmental stage and experimental year, and the complete two-way ANOVA results are provided in **Supplementary Table S1**.

## 3 Results

### 3.1 Effects of straw mulching on early maize growth under different drought intensities

Drought severity markedly affected early maize growth, with leaf area index (LAI), plant height (PH), and dry matter accumulation (DW) generally decreasing as water deficit intensified in both 2025 and 2026 (**Figure 1**). The inhibitory effect of drought was particularly evident at V8. Under straw removal, severe drought (T1) reduced LAI by approximately 39.0%–43.5%, PH by 12.3%–17.4%, and DW by 36.0%–54.8% relative to the well-watered treatment (CK1) across the two years (P < 0.05). Growth traits under moderate drought generally exhibited intermediate values between the well-watered and severe-drought treatments, indicating a consistent increase in growth inhibition with increasing drought severity.

**Figure 1:**
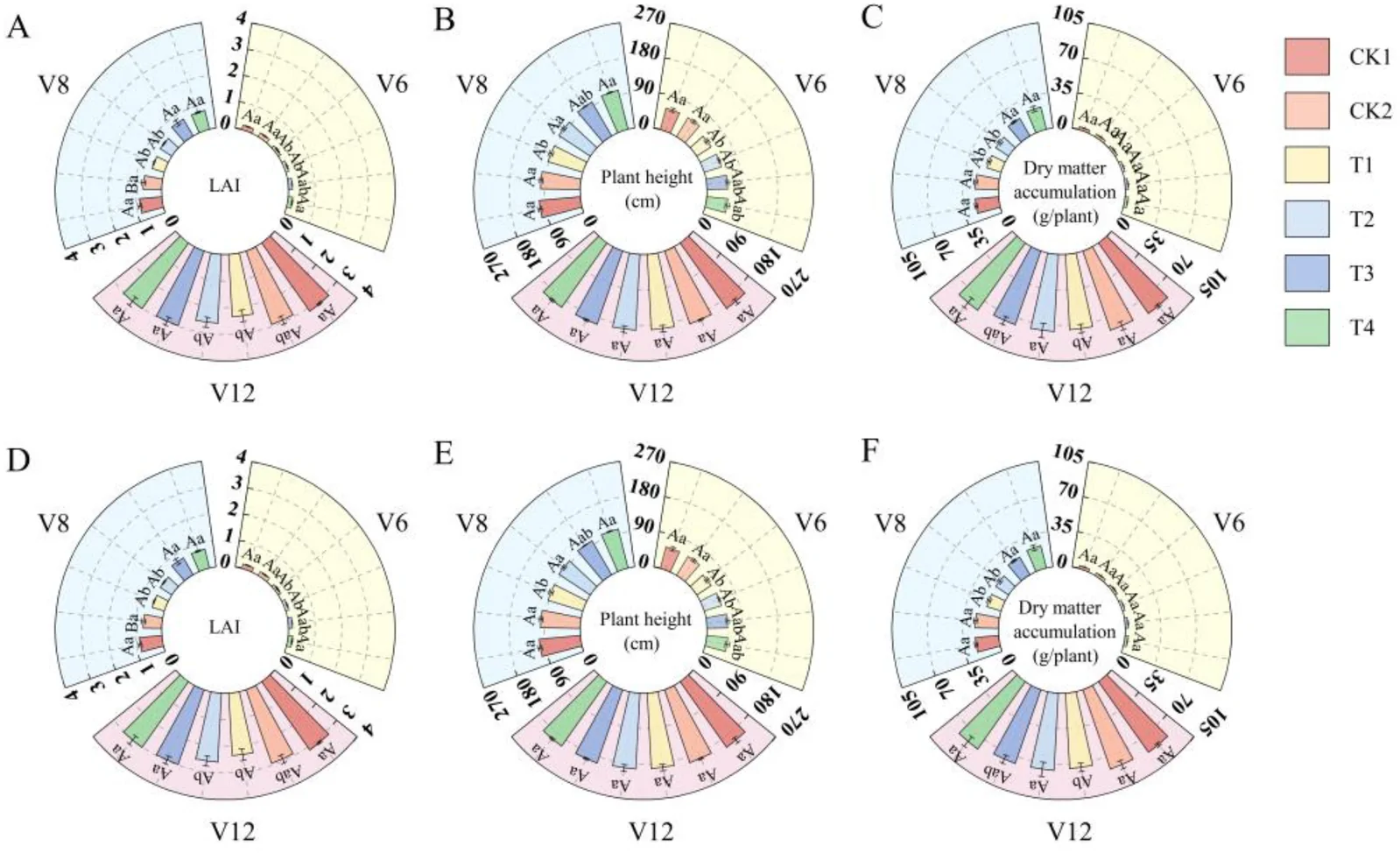
Effects of straw mulching on early maize growth under different drought intensities. **(A–C)** 2025; **(D–F)** 2026. **(A, D)** leaf area index (LAI); **(B, E)**, plant height (PH); **(C, F)** dry matter accumulation (DW). Different uppercase letters indicate significant differences between straw removal and straw mulching under the same irrigation level (P < 0.05), whereas different lowercase letters indicate significant differences among irrigation levels under the same straw treatment (P < 0.05). Error bars represent the standard deviation of three biological replicates.

The response of maize growth to straw mulching showed a similar overall pattern in both years but varied among irrigation levels and traits. Under well-watered conditions, straw mulching did not consistently improve growth, with LAI at V8 being 19.6%–42.6% lower in CK2 than in CK1. Under moderate drought, T4 generally showed higher LAI and DW than T3 at V8, with increases ranging from 0.4% to 9.7% for LAI and from 1.5% to 12.5% for DW across the two years. In contrast, under severe drought, the response to mulching was smaller and less consistent: DW in T2 was only 1.7%–2.3% higher than in T1, while LAI increased by 8.6% in one year but decreased by 16.8% in the other, and most differences between T1 and T2 were not significant (P > 0.05). By V12, the mulching response became more variable among traits. Overall, the two-year results showed that straw mulching produced a more consistent growth-maintaining tendency under moderate drought than under severe drought, although the magnitude and statistical significance of the response differed among growth traits and developmental stages (**Figure 1**). Two-way ANOVA further showed significant irrigation × straw-management interactions for LAI and PH at V8 and for DW at V6 and V8 in both years (P < 0.05), whereas the interactions were not significant for these growth traits at V12 (**Supplementary Table S1**).

### 3.2 Straw mulching maintains leaf photosynthetic performance under drought stress

Drought stress markedly affected leaf photosynthetic performance in maize, with net photosynthetic rate (Pn), stomatal conductance (Gs), intercellular CO_2_ concentration (Ci), and transpiration rate (Tr) generally decreasing as drought severity increased at V6 and V8 in both 2025 and 2026 (**Figure 2**). Under straw removal, severe drought (T1) reduced Pn by 43.1%–45.7%, Gs by 21.6%–28.2%, Ci by 14.9%–20.5%, and Tr by 28.5%–32.7% relative to CK1 at V6 across the two years (P < 0.05). At V8, the corresponding reductions were 25.5%–33.3% for Pn, 39.8%–40.5% for Gs, 15.7%–18.2% for Ci, and 25.2%–28.2% for Tr (P < 0.05). The reductions under moderate drought were generally smaller than those under severe drought, showing a consistent decline in photosynthetic performance as water deficit intensified.

**Figure 2:**
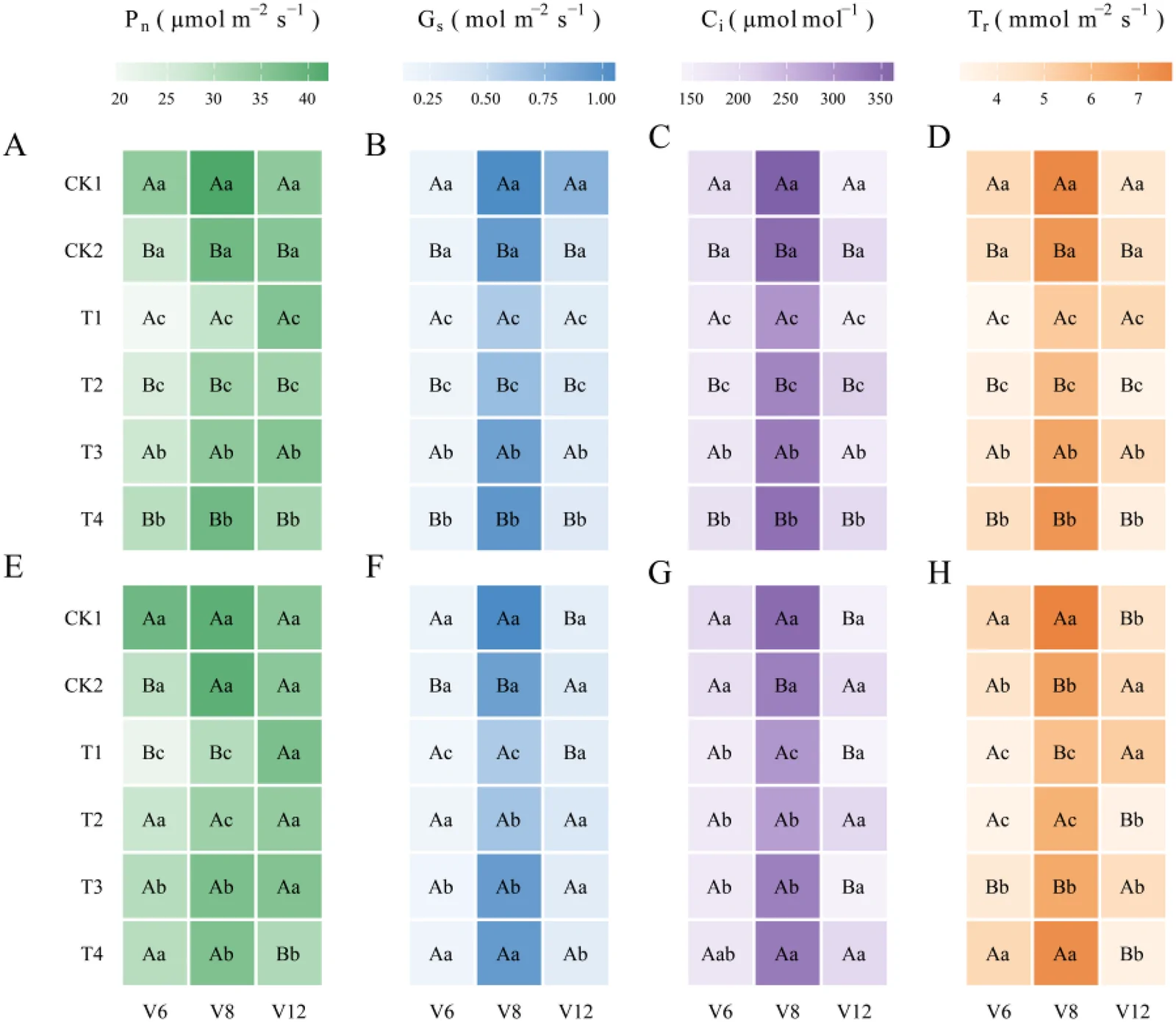
Effects of straw mulching on leaf photosynthetic performance of maize under different drought intensities. **(A–D)** 2025; **(E–H)** 2026. **(A, E)** net photosynthetic rate (Pn); **(B, F)** stomatal conductance (Gs); **(C, G)**, intercellular CO_2_ concentration (Ci); **(D, H)**, transpiration rate (Tr). Different uppercase letters indicate significant differences between straw removal and straw mulching under the same irrigation level (P < 0.05), whereas different lowercase letters indicate significant differences among irrigation levels under the same straw treatment (P < 0.05).

The response of photosynthetic traits to straw mulching showed broadly similar patterns in both years but varied among irrigation levels, developmental stages, and individual parameters. At V6 and V8, several photosynthetic traits were higher under mulching than under the corresponding straw-removal treatment. Under severe drought, mulching increased Pn by up to 31.9%, while increases in Gs and Tr at V8 ranged from 8.2% to 13.6% across the two years. Under moderate drought, T4 showed increases of 4.5%–10.7% in Pn, Gs, Ci, and Tr at V8 relative to T3 in 2025, while Tr increased by 13.4%–22.3% at V6 or V8 in 2026, with several comparisons reaching statistical significance (P < 0.05). However, the magnitude and significance of these responses were not consistent across years and traits. Under well-watered conditions, straw mulching did not consistently improve photosynthetic performance, and CK2 showed lower values of one or more photosynthetic parameters than CK1 at several sampling stages. By V12, the responses became more variable, with mulching generally increasing Gs and Ci but not consistently increasing Pn or Tr. Overall, the two-year results showed that straw mulching more consistently maintained photosynthetic performance at V6 and V8 than at V12, although the response varied among irrigation levels and photosynthetic traits (**Figure 2**).

Further analysis of apparent mesophyll conductance (AMC), water use efficiency (WUE), and stomatal limitation (Ls) showed consistent responses to drought severity across the two years, although the effects of straw mulching varied among developmental stages and irrigation levels (**Figure 3**). At V6 and V8, AMC generally decreased as drought severity increased. Under straw removal, severe drought (T1) reduced AMC by 34.5%–39.8% at V6 and by 18.6%–18.9% at V8 relative to CK1 across the two years. Under drought conditions, AMC was generally higher with straw mulching at these earlier stages. Compared with T1, T2 increased AMC by 26.5%–36.4% at V6 and by 10.1%–15.1% at V8, while AMC in T4 was 5.9%–10.1% higher than that in T3 at V8, with significant differences between the corresponding straw treatments (P < 0.05). By V12, however, this pattern was not maintained, and AMC was lower under mulching than under the corresponding straw-removal treatments, indicating that the AMC response to mulching changed with developmental stage.

**Figure 3:**
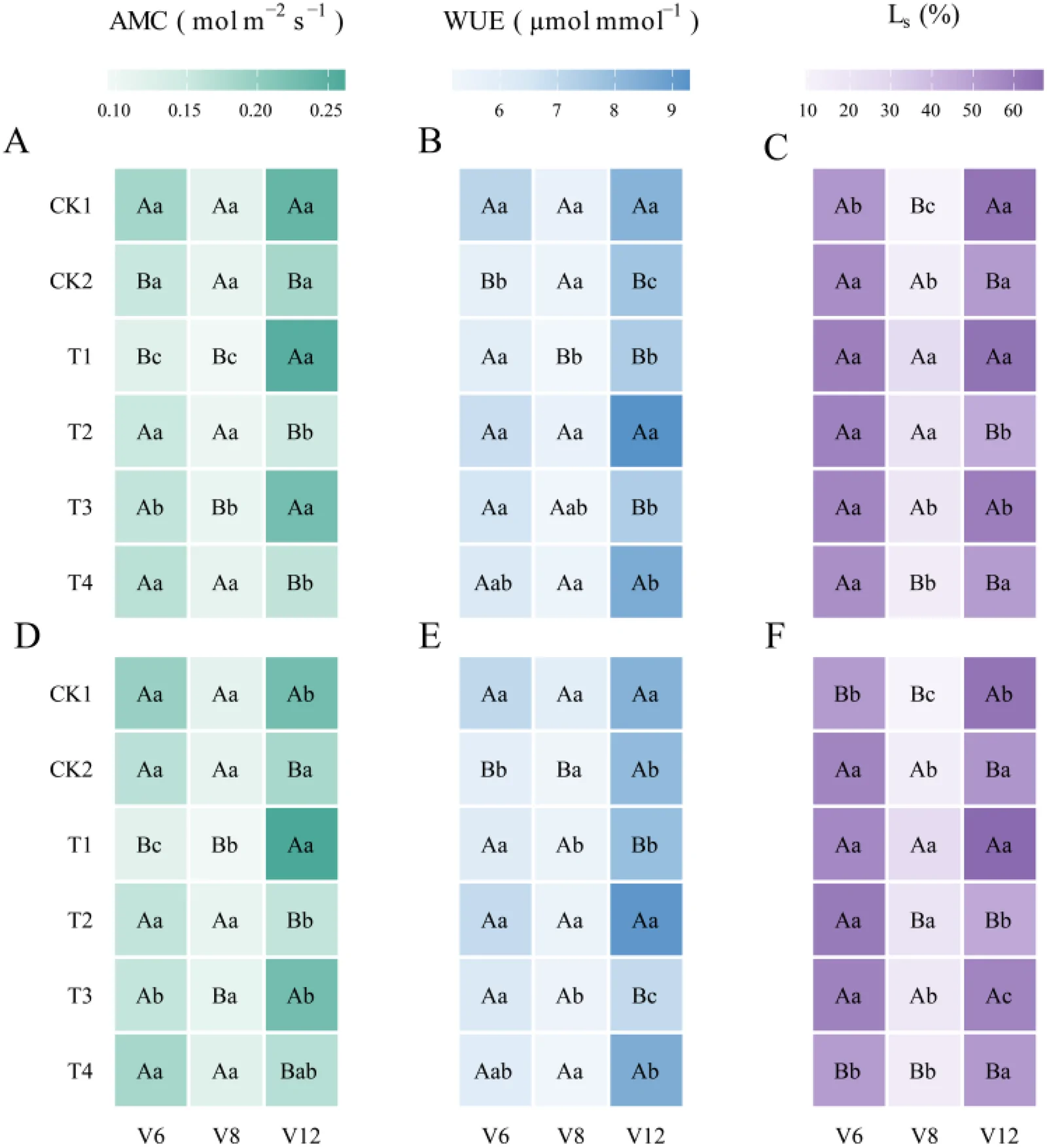
Effects of straw mulching on photosynthetic regulatory traits of maize under different drought intensities. **(A–C)** 2025; **(D–F)** 2026. **(A, D)** apparent mesophyll conductance (AMC); **(B, E)** water use efficiency (WUE); **(C, F)** stomatal limitation (Ls). Different uppercase letters indicate significant differences between straw removal and straw mulching under the same irrigation level (P < 0.05), whereas different lowercase letters indicate significant differences among irrigation levels under the same straw treatment (P < 0.05).

Changes in WUE were relatively small at V6 and V8, whereas clearer differences between straw treatments were observed at V12 in both years. At V12, WUE in T2 was 16.2%–25.2% higher than that in T1, while WUE in T4 was 14.9%–22.1% higher than that in T3 (P < 0.05). In contrast, the response of WUE to mulching at V6 and V8 was comparatively weak and less consistent. Ls increased markedly with drought severity, particularly at V8. Under straw removal, Ls in T1 was approximately 174.0%–175.7% higher than that in CK1, whereas T3 showed increases of 81.4%–94.5% across the two years. Straw mulching was generally associated with lower Ls under drought, especially at V12, where T2 reduced Ls by 28.7%–30.6% relative to T1 and T4 reduced Ls by 11.4%–16.2% relative to T3 (P < 0.05). Overall, the two-year results showed that higher AMC under straw mulching was more evident at V6 and V8, whereas higher WUE and lower Ls were more apparent at V12 (**Figure 3**). Significant irrigation × straw-management interactions were detected for Pn, WUE, and Ls at all developmental stages in both years, whereas the interactions for Gs and AMC occurred mainly at V6 and V8 and those for Tr were primarily detected at V8; in contrast, no significant interaction was observed for Ci (**Supplementary Table S1**).

### 3.3 Straw mulching regulates carbon assimilation under drought stress

Drought stress markedly reduced the activities of the measured carbon-assimilation-related enzymes in maize leaves, with greater reductions generally observed as drought severity increased (**Figure 4**). Under straw removal, severe drought (T1) reduced PC activity by 28.2%–31.9% at V6, 28.2%–28.3% at V8, and 25.0%–29.5% at V12 relative to CK1 across the two years. Under moderate drought (T3), the corresponding reductions were smaller, ranging from 13.8% to 24.7% across developmental stages. RuBPCase activity showed a similar pattern. Relative to CK1, T1 reduced RuBPCase activity by 24.6%–26.0% at V6, 25.8%–31.6% at V8, and 27.8%–33.5% at V12, whereas reductions under T3 ranged from 9.5% to 17.3%. These results indicate that severe drought exerted a stronger inhibitory effect on the measured enzyme activities than moderate drought.

**Figure 4:**
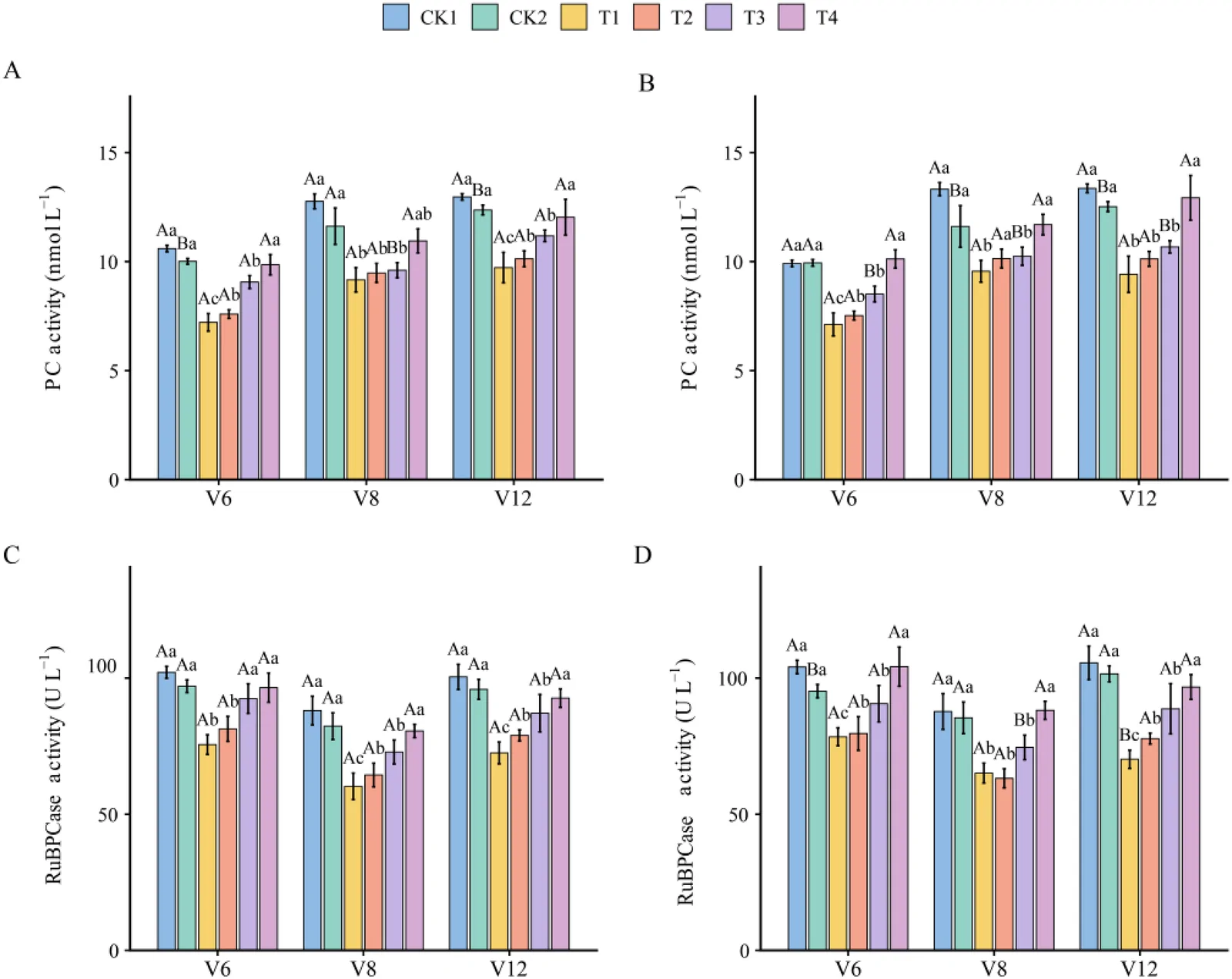
Effects of straw mulching on carbon-assimilation-related enzyme activities in maize leaves under different drought intensities. **(A, C)** 2025; **(B, D)** 2026. **(A, B)** pyruvate carboxylase (PC) activity; **(C, D)** ribulose bisphosphate carboxylase (RuBPCase) activity. Different uppercase letters indicate significant differences between straw removal and straw mulching under the same irrigation level (P < 0.05), whereas different lowercase letters indicate significant differences among irrigation levels under the same straw treatment (P < 0.05).

The response of these enzyme activities to straw mulching showed broadly similar trends across the two years but varied among irrigation levels and developmental stages. Under moderate drought, PC activity in T4 was consistently higher than that in T3, with increases of 8.8%–18.9% at V6, 14.0%–14.2% at V8, and 7.6%–21.1% at V12; significant differences were observed at V8 in 2025 and at all three stages in 2026 (P < 0.05). Under severe drought, PC activity in T2 was only 3.4%–7.5% higher than that in T1, and these differences were not significant (P > 0.05). RuBPCase activity showed a comparable response. Relative to T3, T4 increased RuBPCase activity by 4.4%–15.0% at V6, 10.6%–18.2% at V8, and 6.4%–9.0% at V12, with a significant difference observed at V8 in 2026 (P < 0.05). Under severe drought, differences in RuBPCase activity between T2 and T1 were generally small and nonsignificant, except for a 10.8% increase at V12 in 2026 (P < 0.05). Under well-watered conditions, straw mulching did not consistently increase either enzyme activity. Overall, the two-year results showed that higher enzyme activities under straw mulching were observed more consistently under moderate drought than under severe drought, although the magnitude and significance of the responses varied among developmental stages and enzymes (**Figure 4**). Two-way ANOVA showed significant irrigation × straw-management interactions for both PC and RuBPCase activities at V6, V8, and V12 in both years (P < 0.05; **Supplementary Table S1**), confirming that the responses of these enzyme activities to straw mulching differed among irrigation levels.

### 3.4 Straw mulching modulates osmotic adjustment and membrane lipid peroxidation under drought stress

Drought stress markedly altered osmolyte accumulation and membrane lipid peroxidation in maize leaves, with stronger responses generally observed as water deficit intensified (**Figure 5**). Under straw removal, malondialdehyde (MDA), proline (Pro), soluble sugar (SS), and betaine (BE) contents generally increased with increasing drought severity in both years. Relative to CK1, severe drought (T1) increased MDA content by 18.1%–22.5% at V6, 8.0%–18.8% at V8, and 15.8%–22.8% at V12 across the two years. Pro, SS, and BE showed similar responses, with increases of 15.9%–38.1%, 15.5%–37.3%, and 40.6%–63.3%, respectively, across developmental stages under T1. The corresponding increases under moderate drought (T3) were generally smaller, indicating stronger osmolyte accumulation and membrane lipid peroxidation under severe drought.

**Figure 5:**
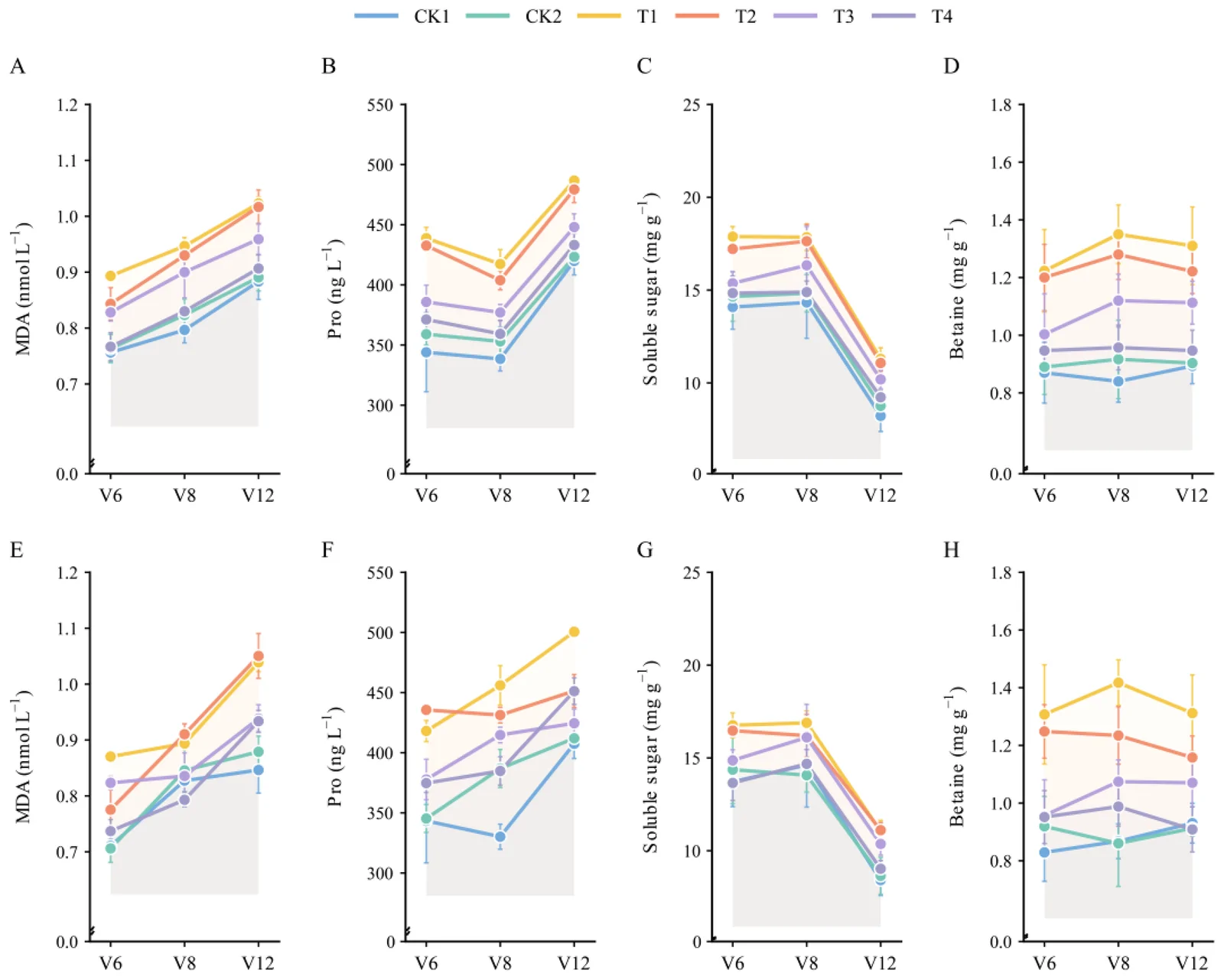
Effects of straw mulching on osmotic adjustment and membrane lipid peroxidation in maize leaves under different drought intensities. **(A–D)** 2025; **(E–H)** 2026. **(A, E)** malondialdehyde (MDA) content; **(B, F)** proline (Pro) content; **(C, G)** soluble sugar (SS) content; **(D, H)** betaine (BE) content. Different uppercase letters indicate significant differences between straw removal and straw mulching under the same irrigation level (P < 0.05), whereas different lowercase letters indicate significant differences among irrigation levels under the same straw management practice (P < 0.05). Error bars represent the standard deviation of three biological replicates.

The response of these traits to straw mulching showed broadly similar trends across the two years but varied among irrigation levels, developmental stages, and individual parameters. Under moderate drought, MDA content in T4 was generally lower than that in T3, with reductions of 7.4%–10.5% at V6, 5.1%–7.8% at V8, and 5.5% at V12, and significant differences were observed at several stages (P < 0.05). Under severe drought, MDA content in T2 was 5.6%–10.9% lower than that in T1 at V6 (P < 0.05), whereas differences at V8 and V12 were comparatively small. Straw mulching was also generally associated with lower Pro, SS, and BE contents under drought. Under moderate drought, reductions in Pro, SS, and BE ranged from 3.3% to 4.7%, 3.4% to 12.9%, and 5.6% to 15.1%, respectively, across stages and years, with significant differences observed for selected traits and stages (P < 0.05). Under severe drought, differences in Pro, SS, and BE between T2 and T1 were generally smaller and less consistent, with significance detected only at specific developmental stages. Overall, the two-year results showed that lower MDA and osmolyte accumulation under straw mulching was observed more consistently under moderate drought than under severe drought, although the magnitude and significance of the response varied among traits and developmental stages (**Figure 5**). The irrigation × straw-management interaction was significant for Pro, BE, and MDA mainly at V6 and V8 in both years, whereas the interaction for SS was less consistent and was primarily detected at V6 (**Supplementary Table S1**), indicating that the mulching response of osmotic and membrane-damage traits was both trait- and stage-dependent.

### 3.5 Straw mulching regulates drought-induced antioxidant defense in maize leaves

Drought stress markedly altered antioxidant enzyme activities in maize leaves, with superoxide dismutase (SOD), peroxidase (POD), and catalase (CAT) activities generally increasing as water deficit intensified in both years (**Figure 6**). Under straw removal, severe drought (T1) increased SOD activity by 14.1%–18.4% across developmental stages in 2025, while POD and CAT activities increased by 14.6%–26.2% and 10.2%–42.0%, respectively. A similar pattern was observed in 2026, with POD activity increasing by 10.4%–32.0% and CAT activity by 15.1%–28.6% at the measured stages relative to CK1. The corresponding increases under moderate drought (T3) were generally smaller, indicating a stronger antioxidant enzyme response under severe drought.

**Figure 6:**
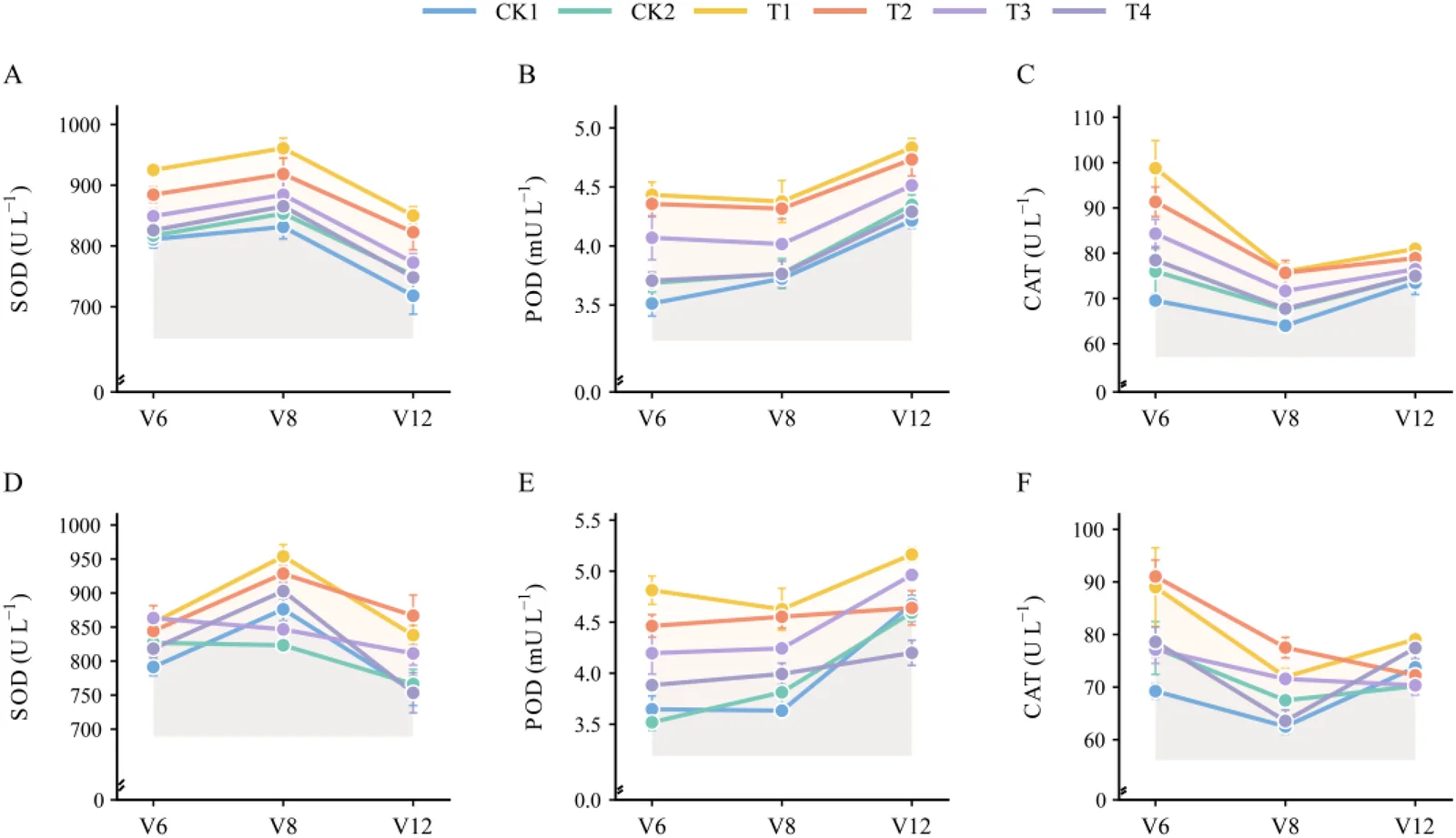
Effects of straw mulching on antioxidant enzyme activities in maize leaves under different drought intensities. **(A–C)** 2025; **(D–F)** 2026. **(A, D)** superoxide dismutase (SOD) activity; **(B, E)** peroxidase (POD) activity; **(C, F)**, catalase (CAT) activity. Different uppercase letters indicate significant differences between straw removal and straw mulching under the same irrigation level (P < 0.05), whereas different lowercase letters indicate significant differences among irrigation levels under the same straw treatment (P < 0.05). Error bars represent the standard deviation of three biological replicates.

The response of antioxidant enzyme activities to straw mulching showed broadly similar trends across the two years but varied among irrigation levels, developmental stages, and individual enzymes. Under severe drought, T2 generally showed lower antioxidant enzyme activities than T1, with reductions in SOD, POD, and CAT ranging from 3.2% to 9.1%, 1.4% to 10.1%, and 0.4% to 7.5%, respectively, across stages and years. Under moderate drought, T4 also generally showed lower POD activity than T3, with reductions of 4.9%–8.9% in 2025 and 5.9%–15.4% in 2026. In contrast, SOD and CAT responses were more variable among developmental stages. For example, at V8 in 2026, SOD activity was 9.1% lower in T2 than in T1 but 6.7% higher in T4 than in T3, while CAT activity also showed alternating increases and decreases among stages. Overall, the two-year results showed that drought consistently increased antioxidant enzyme activities, whereas the response to straw mulching was less uniform and varied among enzymes, developmental stages, and irrigation levels (**Figure 6**). Interaction effects on antioxidant enzyme activities were comparatively limited: significant irrigation × straw-management interactions were mainly detected for SOD and CAT at V6, whereas the interaction for POD was not significant at any developmental stage in either year (**Supplementary Table S1**).

### 3.6 Straw mulching alleviates drought-induced oxidative stress in maize leaves

Drought stress markedly altered reactive-oxygen-related responses and increased H_2_O_2_ accumulation in maize leaves, with stronger responses generally observed as water deficit intensified in both years (**Figure 7**). Under straw removal, the superoxide anion scavenging rate increased with drought severity and was highest under severe drought. Relative to CK1, severe drought (T1) increased the superoxide anion scavenging rate by 79.5%–87.3% at V6, 42.8%–50.1% at V8, and 45.1%–50.7% at V12 across the two years. H_2_O_2_ content showed a similar pattern, increasing by 30.8%–43.1% at V6, 22.3%–24.0% at V8, and 17.5%–21.7% at V12 under T1 relative to CK1. The corresponding increases under moderate drought were generally smaller, indicating greater reactive-oxygen-related responses and H_2_O_2_ accumulation under severe drought.

**Figure 7:**
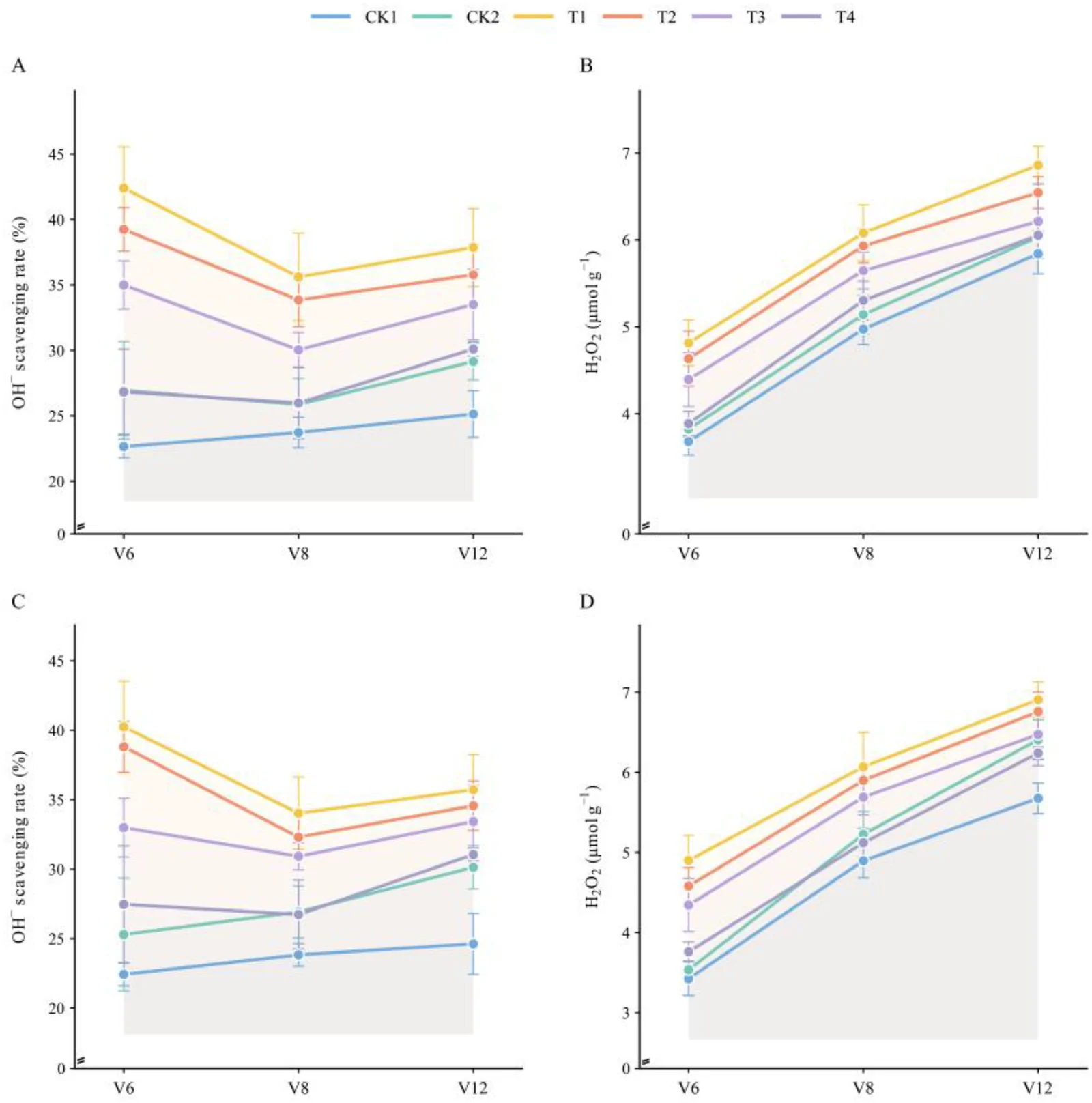
Effects of straw mulching on oxidative stress in maize leaves under different drought intensities. **(A, C)** 2025; **(B, D)** 2026. **(A, B)** superoxide anion scavenging rate; **(C, D)** hydrogen peroxide (H_2_O_2_) content. Different uppercase letters indicate significant differences between straw removal and straw mulching under the same irrigation level (P < 0.05), whereas different lowercase letters indicate significant differences among irrigation levels under the same straw treatment (P < 0.05). Error bars represent the standard deviation of three biological replicates.

The response of these traits to straw mulching showed broadly similar patterns across the two years but varied among irrigation levels and developmental stages. Under moderate drought, T4 reduced the superoxide anion scavenging rate by 16.7%–23.3% at V6, 13.5%–13.6% at V8, and 7.1%–10.2% at V12 relative to T3, with significant differences observed at V6 and/or V8 (P < 0.05). H_2_O_2_ content in T4 was also lower than that in T3, with reductions of 11.5%–13.4% at V6, 6.1%–10.0% at V8, and 2.6%–3.6% at V12 across the two years; significant differences were observed at V6 and V8 in 2026 (P < 0.05). Under severe drought, differences between mulching and straw-removal treatments were comparatively small: T2 reduced the superoxide anion scavenging rate by 3.2%–7.5% and H_2_O_2_ content by 2.2%–6.5% relative to T1, with most differences being nonsignificant (P > 0.05). Overall, the two-year results showed that lower reactive-oxygen-related responses and H_2_O_2_ accumulation under straw mulching were observed more consistently under moderate drought than under severe drought (**Figure 7**). Two-way ANOVA showed significant irrigation × straw-management interactions for H_2_O_2_ content at V6 and V8 in both years, whereas the interaction for superoxide anion scavenging rate was mainly significant at V6 and became nonsignificant at the later stages (**Supplementary Table S1**).

### 3.7 Multivariate analysis of maize responses to straw mulching under different drought intensities

Pearson correlation analysis revealed clear coordinated relationships among growth, photosynthetic, and physiological traits (**Figure 8**). LAI, PH, and DW were positively correlated with each other and were generally positively associated with Pn, Gs, Tr, AMC, PC, and RuBPCase, with a particularly strong correlation between PC and RuBPCase. In contrast, Pro, SS, BE, MDA, O_2_Scav, and H_2_O_2_ were negatively correlated with most growth, photosynthetic, and carbon-assimilation traits, whereas these stress-related variables were generally positively associated with SOD, POD, and CAT. Ls was negatively correlated with Ci but positively associated with several stress-related traits, while WUE showed relatively weak correlations with most variables. Overall, stronger growth and photosynthetic carbon assimilation were associated with lower osmotic and oxidative stress responses, whereas intensified stress responses coincided with reduced growth and carbon-assimilation capacity.

**Figure 8:**
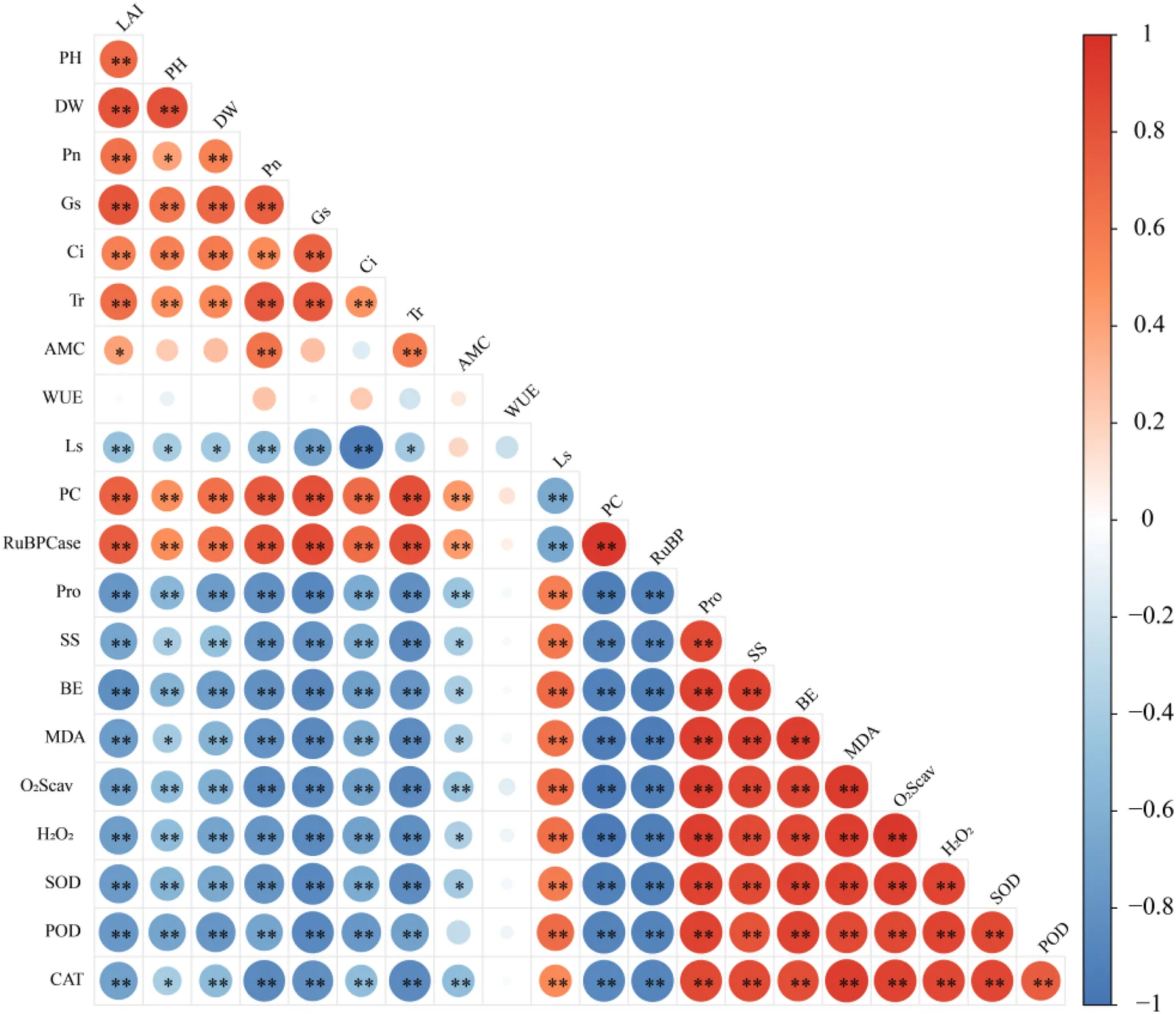
Pearson correlation analysis among maize growth, photosynthetic, and physiological and biochemical traits. Circle size and color intensity represent the strength of correlations, with different colors indicating positive and negative correlations. * and ** indicate significant correlations at P < 0.05 and P < 0.01, respectively. LAI, leaf area index; PH, plant height; DW, dry matter accumulation; Pn, net photosynthetic rate; Gs, stomatal conductance; Ci, intercellular CO_2_ concentration; Tr, transpiration rate; AMC, apparent mesophyll conductance; WUE, water use efficiency; Ls, stomatal limitation; PC, pyruvate carboxylase activity; RuBPCase, ribulose bisphosphate carboxylase activity; Pro, proline; SS, soluble sugar; BE, betaine; MDA, malondialdehyde; O_2_Scav, superoxide anion scavenging rate; H_2_O_2_, hydrogen peroxide; SOD, superoxide dismutase; POD, peroxidase; CAT, catalase.

Principal component analysis further revealed clear differentiation in maize growth and physiological responses among irrigation and straw treatments (**Figure 9**). PC1 and PC2 explained 70.8% and 8.7% of the total variation, respectively, accounting for 79.5% cumulatively. Treatments under 135, 105, and 75 mm irrigation were clearly separated primarily along PC1, indicating that variation in the integrated physiological profile was closely associated with irrigation level. Growth, photosynthetic, and carbon-assimilation traits were generally associated with the negative direction of PC1, whereas MDA, Pro, BE, O_2_Scav, H_2_O_2_, and antioxidant-related traits were mainly associated with the positive direction, consistent with the correlation analysis. Straw removal and straw mulching were also differentiated within each irrigation level. Notably, under 105 mm irrigation, the mulched treatment shifted closer to the 135 mm treatment group, whereas under 75 mm irrigation, the mulched treatment remained clearly separated from the 135 and 105 mm groups. These multivariate patterns indicate that the physiological response to straw mulching differed among irrigation levels, with the mulched treatment under moderate drought exhibiting an integrated physiological profile closer to that under well-watered conditions. The multivariate separation among irrigation and straw treatments was broadly consistent with the trait-specific ANOVA results, in which significant irrigation × straw-management interactions were detected for several key growth, photosynthetic, carbon-assimilation, osmotic, and oxidative-stress traits (**Supplementary Table S1**).

**Figure 9:**
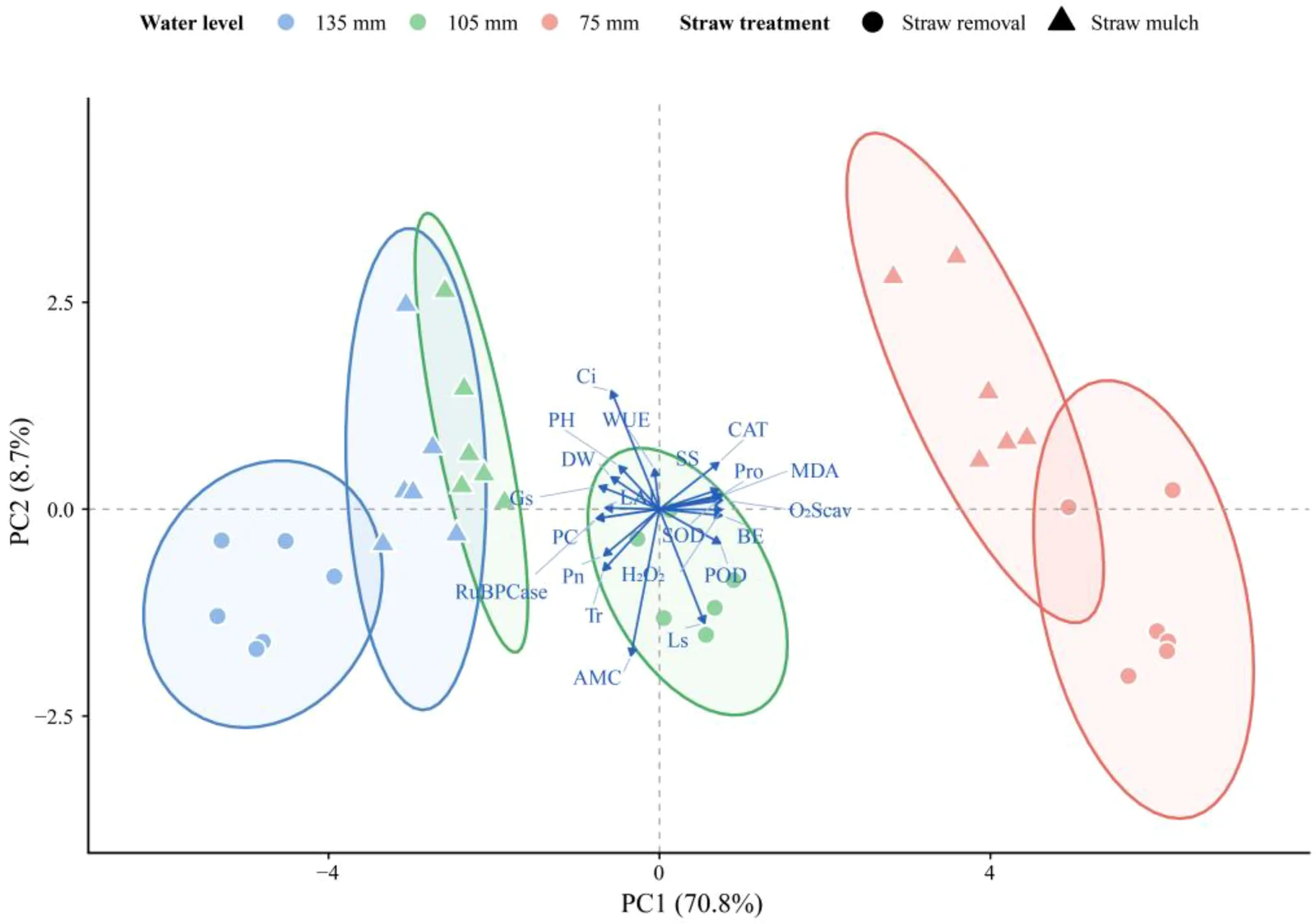
Principal component analysis of maize growth and physiological and biochemical traits under different drought intensities and straw treatments. Different colors represent irrigation levels, whereas circles and triangles indicate straw removal and straw mulching, respectively. Ellipses show the distribution of each treatment group, and arrows indicate the loading direction and contribution of individual traits to the principal components.

## 4 Discussion

### 4.1 The regulation of maize growth and photosynthetic performance by straw mulching depends on drought severity

In this study, increasing water deficit progressively inhibited early maize growth and photosynthetic performance, as indicated by reductions in LAI, PH, DW, Pn, Gs, Ci, Tr, and AMC, with generally greater inhibition under severe drought than under moderate drought. Water deficit restricts leaf expansion and canopy establishment and induces stomatal closure, thereby limiting CO_2_ diffusion into leaves and reducing photosynthetic carbon gain and dry matter accumulation. Consistent with our results, Li et al. (2024) reported that lower soil water availability reduced Pn and Gs and intensified stomatal limitation in maize, ultimately restricting photoassimilate accumulation. In the present study, the drought-induced increase in Ls accompanied by decreases in Pn, Gs, and AMC further indicates that stomatal regulation and restricted CO_2_ diffusion were important components of the early response of maize to water deficit. Moreover, the positive correlations of LAI and DW with most photosynthetic traits support a close coordination between canopy development and leaf photosynthetic capacity. Thus, the transition from moderate to severe drought caused coordinated inhibition of leaf area development, gas exchange, and biomass accumulation rather than isolated changes in individual traits.

Straw mulching modified these drought responses, but its effects varied with drought intensity. Under moderate drought, T4 more consistently maintained LAI, DW, Pn, Gs, Tr, and AMC relative to T3, whereas the differences between T2 and T1 under severe drought were generally smaller and less consistent across years and developmental stages. Previous studies have similarly shown that straw mulching can improve maize growth and photosynthetic performance under water-limited conditions. Zhang et al. (2019) reported increases in LAI, photosynthetic capacity, and aboveground biomass under straw mulching, while Li et al. (2025) found that straw returning improved photosynthetic efficiency and reduced drought-induced losses in dry matter accumulation. One plausible explanation for these responses is that straw mulching can reduce soil evaporation and improve soil water retention, as demonstrated by Amin et al. (2023), who reported increased soil water storage and improved maize productivity under straw mulch. Accordingly, the stronger physiological response to mulching under moderate drought observed in the present study may partly reflect a more favorable root-zone water environment that helped sustain stomatal opening, CO_2_ diffusion, and canopy growth. Under severe drought, however, this potential buffering effect may have been insufficient to offset the greater imposed water deficit, resulting in weaker maintenance of photosynthetic performance and plant growth. Because soil moisture dynamics were not directly quantified in the present study, this interpretation should be regarded as a plausible explanation supported by previous studies rather than a mechanism directly demonstrated here.

Notably, straw mulching did not consistently improve growth or photosynthetic traits under well-watered conditions, as CK2 was lower than CK1 for several traits at specific developmental stages. This indicates that the physiological effects of straw mulching are context dependent rather than universally positive. In addition to influencing soil water status, straw mulch can also modify the soil thermal environment. A multi-year field study in Northeast China showed that straw mulching increased topsoil water content and moderated soil temperature fluctuations, whereas its yield benefit decreased with increasing precipitation and could be constrained by excessive soil cooling (Nie et al., 2026). This hydrothermal trade-off provides a possible explanation for the relatively weak mulching response under sufficient water supply and for some of the stage-dependent responses observed in the present study. Overall, our results show that the growth- and photosynthesis-maintaining effects of straw mulching varied with drought severity, being more consistent under moderate drought and substantially weaker under severe drought. These findings suggest that the physiological benefits of straw mulching operate within a limited range of water deficit rather than increasing continuously as drought intensifies.

### 4.2 The capacity of straw mulching to maintain carbon assimilation under drought is constrained by water deficit severity

Photosynthetic carbon assimilation is a central process linking leaf gas exchange with biomass production and is highly sensitive to water deficit. In the present study, PC and RuBPCase activities decreased progressively with increasing drought severity, with generally greater inhibition under severe drought than under moderate drought. These changes were broadly consistent with the reductions in Pn and AMC, indicating that drought-induced photosynthetic inhibition was not restricted to stomatal regulation and CO_2_ diffusion but also involved biochemical processes associated with carbon assimilation. Previous studies have shown that drought decreases RuBPCase and PEPCase activities in maize, thereby impairing light-energy utilization and carbon assimilation (Hu et al., 2023). Perdomo et al. (2017) further demonstrated that biochemical limitations can become important determinants of photosynthetic inhibition in C_4_ maize under sustained water deficit, indicating that the CO_2_-concentrating mechanism of C_4_ photosynthesis does not completely protect carbon metabolism from drought. More recently, Bellasio et al. (2024) showed that rapid dehydration reduced bundle-sheath conductance in maize, potentially restricting metabolite exchange required for C_4_ photosynthesis and causing feedback inhibition of Rubisco carboxylation. Thus, the stronger reductions in PC and RuBPCase activities observed under severe drought in the present study are consistent with a progressive extension of drought limitation from gas exchange to biochemical processes associated with carbon assimilation.

Straw mulching partially maintained carbon-assimilation capacity under drought, but the magnitude of this response varied with drought severity. Under moderate drought, PC activity in T4 was consistently higher than that in T3 across most developmental stages and years, and RuBPCase generally showed a similar pattern. In contrast, the increases in T2 relative to T1 under severe drought were smaller and mostly nonsignificant. These results indicate that the physiological benefits of straw mulching under moderate drought extended beyond gas exchange to carbon-assimilation-related enzyme activities, whereas these benefits became substantially weaker as water deficit intensified. Li et al. (2025) reported that straw returning alleviated drought-induced reductions in maize gas exchange and photosynthetic efficiency and reduced losses in dry matter accumulation. Using ¹³C pulse labeling, Wang et al. (2022) further demonstrated that straw return could enhance the assimilation and subsequent allocation of newly fixed photosynthetic carbon in maize. Although straw management practices and water conditions differed among these studies, the available evidence suggests that mulch-induced improvements in the root-zone environment may contribute to the maintenance of photosynthetic carbon acquisition under water-limited conditions.

One plausible explanation for the stronger response under moderate drought is that straw mulching may reduce soil evaporation and slow soil-water depletion, thereby helping to maintain a more favorable root-zone water environment. Under moderate water deficit, such effects may partially support stomatal opening, CO_2_ supply, and carbon-assimilation processes. Under more severe drought, however, this potential buffering effect may be insufficient to offset the greater imposed water deficit, resulting in persistent inhibition of photosynthetic metabolism. Consistent with this interpretation, Lu et al. (2026) reported that the combination of straw mulching with moderate deficit irrigation provided a favorable balance between water saving and maintenance of maize productivity. Because soil moisture dynamics were not directly quantified in the present study, however, this explanation should be regarded as a plausible mechanism supported by previous studies rather than direct evidence from our experiment.

Notably, PC and RuBPCase activities under well-watered conditions were not consistently higher in CK2 than in CK1, indicating that straw mulching does not universally stimulate carbon-assimilation-related processes. Taken together with the gas-exchange responses, our results show that straw mulching under moderate drought more consistently maintained both CO_2_ exchange and carbon-assimilation-related enzyme activities, whereas these responses were substantially weaker under severe drought. This pattern suggests that the physiological effectiveness of straw mulching is closely associated with drought severity, although the underlying soil-water mechanism requires direct verification through measurements of soil moisture dynamics.

### 4.3 Straw mulching maintains cellular homeostasis by modulating drought-induced osmotic and oxidative stress

Plants respond to water deficit through coordinated osmotic adjustment and antioxidant defense to maintain cellular water status and redox homeostasis. In the present study, increasing drought severity generally enhanced the accumulation of Pro, SS, and BE and increased MDA and H_2_O_2_ contents, while the superoxide anion scavenging rate and the activities of SOD, POD, and CAT also increased to varying degrees. These responses were generally stronger under severe drought than under moderate drought, indicating that increasing water deficit simultaneously stimulated osmotic adjustment, intensified oxidative stress and membrane lipid peroxidation, and activated antioxidant defense. Eskikoy and Kutlu (2024) similarly showed that Pro, MDA, H_2_O_2_, and antioxidant enzyme responses in maize differed markedly between moderate and severe drought, demonstrating a strong dependence of these physiological responses on drought severity. Gao et al. (2024) also reported enhanced Pro accumulation and activation of SOD and POD in drought-stressed maize seedlings, followed by readjustment of these stress responses after rewatering. Thus, the greater accumulation of osmolytes, MDA, and H_2_O_2_ and the stronger antioxidant responses observed under severe drought in the present study are consistent with a more intense physiological response to water and oxidative stress rather than simply indicating greater stress tolerance.

The accumulation of Pro, SS, and BE contributes to osmotic adjustment by lowering cellular osmotic potential, maintaining water uptake, and protecting cellular structures. Pro can additionally contribute to membrane stabilization and redox regulation. Khan et al. (2025) showed that Pro application improved maize water status and growth under drought while reducing H_2_O_2_ and MDA accumulation through coordinated regulation of osmoprotection and antioxidant defense, highlighting the close association between osmotic adjustment and oxidative stress regulation. In the present study, however, straw mulching did not further stimulate the accumulation of Pro, SS, and BE under drought; instead, these osmolytes were generally lower in mulched plants, particularly in T4 relative to T3 under moderate drought. Together with the concurrent reductions in MDA and H_2_O_2_, this pattern suggests that mulched plants experienced a lower overall physiological stress load and therefore required less intensive osmotic adjustment. Thus, the relatively lower concentrations of Pro, SS, and BE under mulching, together with reduced MDA and H_2_O_2_, are more consistent with a less stressful cellular environment than with a weakening of osmotic adjustment capacity.

A similar pattern was observed for oxidative stress and antioxidant defense. Under moderate drought, straw mulching generally reduced MDA and H_2_O_2_ accumulation, attenuated the drought-induced superoxide-scavenging response, and resulted in lower SOD, POD, or CAT activities at several developmental stages compared with straw removal. Lower antioxidant enzyme activities under mulching therefore do not necessarily indicate impaired antioxidant defense; rather, they may reflect a lower oxidative burden and consequently a reduced requirement for sustained enzymatic defense. In a semi-arid maize production system, Yuan et al. (2023) found that a water-conserving cultivation system involving straw mulch improved soil water storage, reduced MDA, H_2_O_2_, and O_2_^-^ accumulation, and modified SOD and CAT responses. Zhao et al. (2024) likewise reported that mulching reduced MDA accumulation while improving photosynthetic performance and antioxidant status in rainfed maize. Moreover, Guo et al. (2024) observed that straw return simultaneously decreased MDA content and the activities of several antioxidant enzymes under another abiotic-stress environment, supporting the interpretation that lower antioxidant enzyme activity can accompany reduced oxidative damage rather than weakened stress defense.

Importantly, the extent to which straw mulching modulated osmotic and oxidative responses varied with drought severity. Under moderate drought, mulching produced relatively consistent reductions in MDA and H_2_O_2_ and moderated the accumulation of Pro, SS, and BE and the activation of antioxidant defenses. Under severe drought, however, differences between T2 and T1 became much smaller, and the effects of mulching on H_2_O_2_, superoxide anion scavenging, and most antioxidant enzymes were weak. One plausible explanation is that straw mulching may have improved the root-zone environment under moderate drought, thereby reducing the effective water and oxidative stress experienced by plants and limiting the need for intensive osmotic and antioxidant responses. Under severe drought, however, this potential buffering effect may have been insufficient to offset the greater imposed water deficit, leaving plants in a more strongly activated stress-defense state. Because soil moisture dynamics were not directly quantified in the present study, this interpretation should be regarded as a plausible mechanism supported by previous studies rather than direct evidence from our experiment. Overall, the results indicate that straw mulching more consistently contributed to cellular homeostasis under moderate drought, whereas these physiological benefits became substantially weaker under severe drought.

### 4.4 Multivariate analyses reveal integrated physiological responses to straw mulching under different drought intensities

Correlation and principal component analyses further revealed the integrated physiological responses of maize to straw mulching under different drought intensities. LAI, PH, and DW were generally positively associated with photosynthetic and carbon-assimilation traits, whereas Pro, SS, BE, MDA, O_2_Scav, and H_2_O_2_ were negatively associated with growth and carbon-assimilation traits. This pattern indicates a coordinated shift from growth and carbon acquisition toward osmotic adjustment and oxidative defense as water deficit intensified. PCA further supported this integrated response, with PC1 and PC2 together explaining 79.5% of the total variation and treatments under different irrigation levels separating mainly along PC1. This separation indicates that variation in the overall physiological profile was closely associated with irrigation level, although PCA itself does not formally partition the variance attributable to individual experimental factors.

Under moderate drought, the mulched treatment shifted toward the well-watered treatments in the PCA space, whereas under severe drought the mulched treatment remained clearly separated from the well-watered group. This multivariate pattern was consistent with the responses observed for growth, photosynthesis, carbon-assimilation-related enzyme activities, osmotic adjustment, and oxidative stress. Nie et al. (2026) demonstrated that the effectiveness of straw mulching can be influenced by the balance between its soil-water conservation and thermal effects, while Lu et al. (2026) reported that moderate deficit irrigation combined with straw mulching achieved a more favorable balance between water saving and maize productivity than severe deficit irrigation. Taken together, these findings suggest that the physiological benefits of straw mulching vary with drought severity and are more consistently expressed under moderate water deficit, whereas they become substantially weaker under severe drought. The soil-water and thermal processes reported in previous studies provide plausible explanations for this pattern, but these mechanisms were not directly quantified in the present experiment.

Several limitations of this study should be acknowledged. First, the present experiment focused on early vegetative growth from V6 to V12; therefore, the observed physiological responses should not be directly extrapolated to whole-season crop performance or final grain yield. Second, although the treatment responses observed here are consistent with the soil-water-conservation and hydrothermal effects of straw mulching reported in previous studies, soil moisture and soil temperature dynamics were not directly quantified in the present study. Therefore, these processes should be regarded as plausible explanations rather than mechanisms directly demonstrated by our experiment. Future studies should integrate continuous monitoring of soil water and temperature with whole-season measurements of growth, water use, and grain yield to determine whether the drought-severity-dependent physiological responses observed during early growth translate into sustained agronomic benefits.

## 5 Conclusions

This study demonstrates that the physiological effectiveness of straw mulching during early maize growth varied markedly with drought severity. Increasing water deficit progressively inhibited LAI, PH, DW, photosynthetic performance, and carbon-assimilation capacity, while enhancing osmotic adjustment, membrane lipid peroxidation, and oxidative-stress responses. Straw mulching partially maintained maize growth, photosynthetic performance, and carbon assimilation and contributed to cellular homeostasis by reducing MDA and H_2_O_2_ accumulation and modulating osmotic and antioxidant responses. These beneficial effects were more consistent under moderate drought but became substantially weaker and less consistent under severe drought. Correlation and PCA further showed clear associations among irrigation level, straw management, and the integrated physiological responses of maize, with the mulched treatment under moderate drought exhibiting a physiological profile closer to that under well-watered conditions. Overall, straw mulching provided more consistent physiological protection under moderate drought, whereas its capacity to alleviate stress was markedly reduced under severe drought. These findings indicate that the effectiveness of straw mulching is constrained by drought severity and suggest that, under severe water deficit, straw mulching alone may be insufficient to maintain early maize physiological performance. Future water-management strategies should therefore consider integrating straw mulching with supplemental irrigation or other complementary practices under severe drought conditions.

## Data Availability

The original contributions presented in the study are included in the article/**Supplementary Material**. Further inquiries can be directed to the corresponding authors.
