# Supplementary material for: Drought severity determines the effectiveness of straw mulching in alleviating water stress during early maize growth
Source: Front Plant Sci. 2026 Oct 22;17:1983668. doi: 10.3389/fpls.2026.1983668 (PMC13735722; doi:10.3389/fpls.2026.1983668)
Supplement: Supplementary file 1 [file Table1.docx]

Supplementary Material

Table S1. Two-way ANOVA results for the effects of irrigation level (I), straw management (S), and their interaction (I × S) on maize traits at V6, V8, and V12 in 2025 and 2026.

| Trait | 2025 | | | | | | | | | 2026 | | | | | | | | | |
| --- | --- | --- | --- | --- | --- | --- | --- | --- | --- | --- | --- | --- | --- | --- | --- | --- | --- | --- | --- |
|  | V6 | | | V8 | | | V12 | | | V6 | | | V8 | | | V12 | | | |
|  | I | S | I × S | I | S | I × S | I | S | I × S | I | S | I × S | I | S | I × S | I | S | I × S |  |
| LAI | ** | * | ns | ** | * | * | ** | * | ns | ** | * | ns | ** | * | * | ** | * | ns |  |
| PH | ** | * | ns | ** | * | * | ** | * | ns | ** | * | ns | ** | * | * | ** | * | ns |  |
| DW | ** | * | * | ** | ** | * | ** | * | ns | ** | * | * | ** | ** | * | ** | * | ns |  |
| Pn | ** | ** | * | ** | ** | * | ** | ** | * | ** | ** | * | ** | ** | * | ** | ** | * |  |
| Gs | ** | * | * | ** | ** | * | ** | * | ns | ** | * | * | ** | ** | * | ** | * | ns |  |
| Ci | ** | * | ns | ** | * | ns | ** | * | ns | ** | * | ns | ** | * | ns | ** | * | ns |  |
| Tr | ** | * | ns | ** | * | * | ** | * | ns | ** | * | ns | ** | * | * | ** | * | ns |  |
| AMC | ** | * | * | ** | * | * | ** | * | ns | ** | * | * | ** | * | * | ** | * | ns |  |
| WUE | ** | * | * | ** | * | * | ** | * | * | ** | * | * | ** | * | * | ** | * | * |  |
| Ls | ** | * | * | ** | * | * | ** | * | * | ** | * | * | ** | * | * | ** | * | * |  |
| PC | ** | * | * | ** | * | * | ** | * | * | ** | * | * | ** | * | * | ** | * | * |  |
| RuBPCase | ** | * | * | ** | * | * | ** | * | * | ** | * | * | ** | * | * | ** | * | * |  |
| Pro | ** | * | * | ** | * | * | ** | * | ns | ** | * | * | ** | * | * | ** | * | ns |  |
| SS | ** | * | * | ** | * | ns | ** | * | ns | ** | * | * | ** | * | ns | ** | * | ns |  |
| BE | ** | * | * | ** | * | * | ** | * | ns | ** | * | * | ** | * | * | ** | * | ns |  |
| MDA | ** | * | * | ** | * | * | ** | * | ns | ** | * | * | ** | * | * | ** | * | ns |  |
| O₂Scav | ** | * | * | ** | * | ns | ** | * | ns | ** | * | * | ** | * | ns | ** | * | ns |  |
| H₂O₂ | ** | * | * | ** | * | * | ** | * | ns | ** | * | * | ** | * | * | ** | * | ns |  |
| SOD | ** | * | * | ** | * | ns | ** | * | ns | ** | * | * | ** | * | ns | ** | * | ns |  |
| POD | ** | * | ns | ** | * | ns | ** | * | ns | ** | * | ns | ** | * | ns | ** | * | ns |  |
| CAT | ** | * | * | ** | * | ns | ** | * | ns | ** | * | * | ** | * | ns | ** | * | ns |  |
